# Supplementary material for: The prevalence of SARS-CoV-2 infection and other public health outcomes during the BA.2/BA.2.12.1 surge, New York City, April–May 2022
Source: Commun Med (Lond). 2023 Jun 30;3:92. doi: 10.1038/s43856-023-00321-w (PMC10313770; doi:10.1038/s43856-023-00321-w)
Supplement: Supplementary file 1 — Reporting Summary [file 43856_2023_321_MOESM1_ESM.pdf]

nature portfolio

Corresponding author(s): Denis Nash

Last updated by author(s): 2023-19-1

Reporting Summary

Nature Portfolio wishes to improve the reproducibility of the work that we publish. This form provides structure for consistency and transparency in reporting. For further information on Nature Portfolio policies, see our Editorial Policies and the Editorial Policy Checklist.

Statistics

For all statistical analyses, confirm that the following items are present in the figure legend, table legend, main text, or Methods section.

n/a	Confirmed	
☒	☐	The exact sample size (*n*) for each experimental group/condition, given as a discrete number and unit of measurement
☒	☐	A statement on whether measurements were taken from distinct samples or whether the same sample was measured repeatedly
☐	☒	The statistical test(s) used AND whether they are one- or two-sided<br>*Only common tests should be described solely by name; describe more complex techniques in the Methods section.*
☐	☒	A description of all covariates tested
☒	☐	A description of any assumptions or corrections, such as tests of normality and adjustment for multiple comparisons
☐	☒	A full description of the statistical parameters including central tendency (e.g. means) or other basic estimates (e.g. regression coefficient) AND variation (e.g. standard deviation) or associated estimates of uncertainty (e.g. confidence intervals)
☐	☒	For null hypothesis testing, the test statistic (e.g. *F*, *t*, *r*) with confidence intervals, effect sizes, degrees of freedom and *P* value noted<br>*Give P values as exact values whenever suitable.*
☒	☐	For Bayesian analysis, information on the choice of priors and Markov chain Monte Carlo settings
☒	☐	For hierarchical and complex designs, identification of the appropriate level for tests and full reporting of outcomes
☒	☐	Estimates of effect sizes (e.g. Cohen's *d*, Pearson's *r*), indicating how they were calculated

Our web collection on statistics for biologists contains articles on many of the points above.

Software and code

Policy information about availability of computer code

Data collection	N/A
Data analysis	N/A

For manuscripts utilizing custom algorithms or software that are central to the research but not yet described in published literature, software must be made available to editors and reviewers. We strongly encourage code deposition in a community repository (e.g. GitHub). See the Nature Portfolio guidelines for submitting code & software for further information.

Data

Policy information about availability of data

All manuscripts must include a data availability statement. This statement should provide the following information, where applicable:

- Accession codes, unique identifiers, or web links for publicly available datasets
- A description of any restrictions on data availability
- For clinical datasets or third party data, please ensure that the statement adheres to our policy

Deidentified survey data are available on request. SARS-CoV-2 routine testing and case surveillance data and SARS-CoV-2 wastewater surveillance data are publicly available at the following links:
1. Daily data on SARS-CoV-2 cases, hospitalizations, and deaths in NYC https://github.com/nychealth/coronavirus-data/tree/master/trends#data-by-daycsv

2. SARS-CoV-2 concentrations measured in NYC Wastewater. NYC Open Data https://data.cityofnewyork.us/Health/SARS-CoV-2-concentrations-measured-in-NYC-Wastewat/f7dc-2q9f/data

Human research participants

Policy information about studies involving human research participants and Sex and Gender in Research.

Reporting on sex and gender	Survey participants could identify as male, female, or non-binary. We report this as gender.
Population characteristics	We report on a representative sample of the NYC adult population over age 18.
Recruitment	Sampling Frame<br>A sampling frame of 5,580,901 residents of New York City was used of which 2,185,433 have mobile numbers with an additional 1,539,793 landlines. Two stratified proportionate randomized population-based samples were drawn for this study, n=45,000 mobile numbers and n=60,000<br>landlines. A total sample of n=1,030 was utilized with a +/- 3 percent margin of error.<br>Multi-mode data collection design<br>Short message service (SMS) aka text messages were sent using SMS platform. The respondents were sent a personalized first name text message which included a link to the survey and an opt-out option. The respondents had the option to reply to the SMS text with any queries and the survey was available in both English and Spanish. Data was verified by IP address and scrubbed against the original survey sample.<br>Interactive voice response (IVR) aka robo-poll messages were sent to landlines. The respondents were able to answer the survey questions using the touch tone keypad on their phones in either English or Spanish.<br>Data were collected on May 7 and 8, 2022.<br><br>Our survey estimates of SARS-CoV-2 prevalence and provider testing are subject to selection and information biases. In terms of selection bias, our survey estimates may be biased due to non-response if those who responded differ from non-respondents. We could not correct for this bias; however, characteristics of survey respondents did not differ substantially from that of the adult NYC population (Appendix 1 in Supplementary Materials).
Ethics oversight	The study was approved by the IRB of the City University of New York (CUNY)

Note that full information on the approval of the study protocol must also be provided in the manuscript.

Field-specific reporting

Please select the one below that is the best fit for your research. If you are not sure, read the appropriate sections before making your selection.

☐ Life sciences ☒ Behavioural & social sciences ☐ Ecological, evolutionary & environmental sciences

For a reference copy of the document with all sections, see nature.com/documents/nr-reporting-summary-flat.pdf

Behavioural & social sciences study design

All studies must disclose on these points even when the disclosure is negative.

Study description	Quantitative, cross-sectional population-based survey
Research sample	A representative sample of the adult population of NYC.<br><br>SARS-CoV-2 routine testing and case surveillance data and SARS-CoV-2 wastewater surveillance data are publicly available at the following links:<br>1. Daily data on SARS-CoV-2 cases, hospitalizations, and deaths in NYC https://github.com/nychealth/coronavirus-data/tree/master/trends#data-by-daycsv<br><br>2. SARS-CoV-2 concentrations measured in NYC Wastewater. NYC Open Data https://data.cityofnewyork.us/Health/SARS-CoV-2-concentrations-measured-in-NYC-Wastewat/f7dc-2q9f/data
Sampling strategy	A sampling frame of 5,580,901 residents of New York City was used of which 2,185,433 have mobile numbers with an additional 1,539,793 landlines. Two stratified proportionate randomized population-based samples were drawn for this study, n=45,000 mobile numbers and n=60,000<br>landlines. A total sample of n=1,030 was utilized with a +/- 3 percent margin of error.
Data collection	Multi-mode data collection design

Data collection	Short message service (SMS) aka text messages were sent using SMS platform. The respondents were sent a personalized first name text message which included a link to the survey and an opt-out option. The respondents had the option to reply to the SMS text with any queries and the survey was available in both English and Spanish. Data was verified by IP address and scrubbed against the original survey sample.<br>Interactive voice response (IVR) aka robo-poll messages were sent to landlines. The respondents were able to answer the survey questions using the touch tone keypad on their phones in either English or Spanish.<br>Data were collected on May 7 and 8, 2022.
Timing	Data were collected on May 7 and 8, 2022.
Data exclusions	No exclusions
Non-participation	N/A
Randomization	N/A

Reporting for specific materials, systems and methods

We require information from authors about some types of materials, experimental systems and methods used in many studies. Here, indicate whether each material, system or method listed is relevant to your study. If you are not sure if a list item applies to your research, read the appropriate section before selecting a response.

Materials & experimental systems

n/a	Involved in the study
☒	☐ Antibodies
☒	☐ Eukaryotic cell lines
☒	☐ Palaeontology and archaeology
☒	☐ Animals and other organisms
☒	☐ Clinical data
☒	☐ Dual use research of concern

Methods

n/a	Involved in the study
☒	☐ ChIP-seq
☒	☐ Flow cytometry
☒	☐ MRI-based neuroimaging
