# Supplementary material for: The prevalence of SARS-CoV-2 infection and other public health outcomes during the BA.2/BA.2.12.1 surge, New York City, April–May 2022
Source: Commun Med (Lond). 2023 Jun 30;3:92. doi: 10.1038/s43856-023-00321-w (PMC10313770; doi:10.1038/s43856-023-00321-w)
Supplement: Supplementary file 3 — Description of Additional Supplementary Files [file 43856_2023_321_MOESM3_ESM.pdf]

Description of Additional Supplementary Files

File name: Supplementary Data 1
Description: Characteristics of survey respondents by testing status, NYC April-May 2022.
