# Supplementary material for: The prevalence of SARS-CoV-2 infection and other public health outcomes during the BA.2/BA.2.12.1 surge, New York City, April–May 2022
Source: Commun Med (Lond). 2023 Jun 30;3:92. doi: 10.1038/s43856-023-00321-w (PMC10313770; doi:10.1038/s43856-023-00321-w)
Supplement: Supplementary file 5 — Supplementary Information [file 43856_2023_321_MOESM5_ESM.pdf]

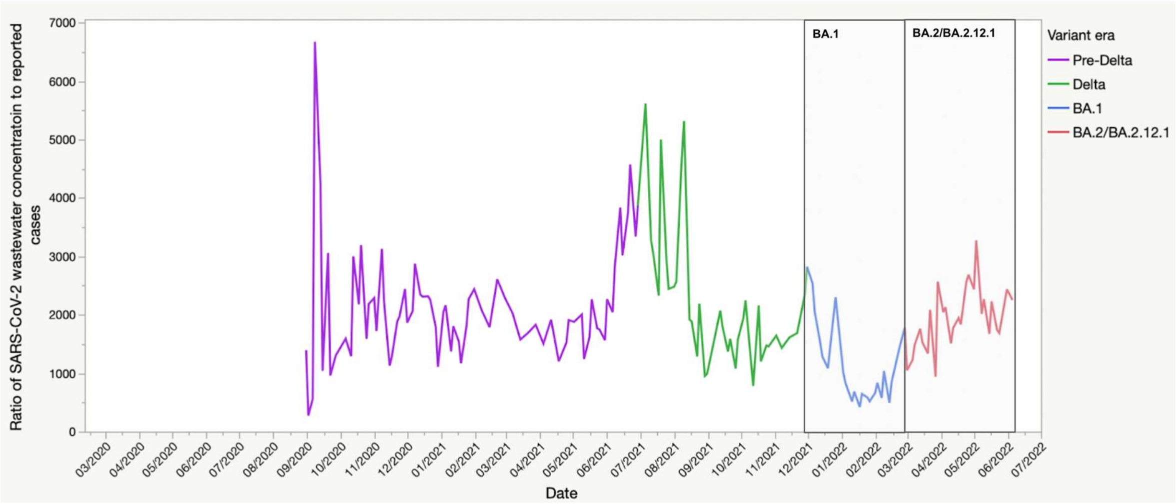


**Supplementary Figure 1. The ratio of SARS-CoV-2 wastewater concentration to reported cases.** Variant eras for NYC were approximated based on the timing of peaks and troughs in COVID-related hospitalizations in NYC as follows: 1 December 2021-1 March 2022 (BA.1) and 1 March 2022-6 June 2022 (BA.2)

## Supplementary Methods

### *Survey design*

Sampling Frame

A sampling frame of 5,580,901 residents of New York City was used of which 2,185,433 have mobile numbers with an additional 1,539,793 landlines. Two stratified proportionate randomized population-based samples were drawn for this study, n=45,000 mobile numbers and n=60,000 landlines. A total sample of n=1,030 was utilized with a +/- 3 percent margin of error.

Multi-mode data collection design

Short message service (SMS) aka text messages were sent using SMS platform. The respondents were sent a personalized first name text message which included a link to the

survey and an opt-out option. The respondents had the option to reply to the SMS text with any queries and the survey was available in both English and Spanish. Data was verified by IP address and scrubbed against the original survey sample.

Interactive voice response (IVR) aka robo-poll messages were sent to landlines. The respondents were able to answer the survey questions using the touch tone keypad on their phones in either English or Spanish.

Data were collected on May 7 and 8, 2022.

Weighting

The survey was weighted using an iterative weighting method (raking) to marginal proportions of race, ethnicity, age, gender and education by borough. The sum of the weights equals the sample population (n=1030). The demographic weights were developed based on the American Community Survey ANNUAL ESTIMATES OF THE RESIDENT POPULATION: APRIL 1, 2010 TO JULY 1, 2019 - FOR FULL ESTIMATES DETAIL, VISIT: https://www.census.gov/programs-surveys/popest.html. Since substantial numbers of New Yorkers moved temporarily or permanently during the pandemic, the ACS data from 2019 is likely less representative of the NYC population in 2022.

The weights were based on respondent self-identified sex, educational attainment, age, ethnicity/race and region. The inference population is 6,740,580 million adult NYC residents.

## Supplementary Results

*Response rates*

In the table below, we present the response rates for our survey by modality. For context, we also present response rates and participation rates for the same modalities for New York City's 2020 Community Health Survey (CHS) and the NYC Health Opinion Poll (HOP) in Q1 (HOP1) and Q2 (HOP2) in 2019. While our response and participation rates are low, population-based surveys, including those in NYC, have seen increasingly low response rates (Table). The higher response rates for the CHS survey relative to our BA.2 survey could be due to publicity and outreach efforts that are conducted by the NYC Department of Health in order to increase participation. Lower response rates in our survey could be due to the fact that it was conducted during a COVID surge when people may have been less likely to be available to participate in surveys. Importantly, population-based surveys in the US have been seeing increasingly low response rates but as a recent paper by the Pew Research Center notes, they can still provide accurate data with low response rates. To assess the impact of non-response on study validity, studies that have examined non-response in surveys suggest that response rates are a poor indicator of non-response bias and data quality.

Supplementary Table 1

| | NYC BA.2 Survey (current survey) | | | 2020 Community Health Survey (CHS) | | | NYC HOP-1 (2019) | NYC HOP-2 (2019) |
|---|---|---|---|---|---|---|---|---|
| **Modality** | **Landline** | **Cell** | **Combined** | **Landline** | **Cell** | **Combined** | **Online opt-in panel** | **Online opt-in panel** |
| **Response rate (%)** | 1.3 | 0.6 | 1.2 | 6 | 7.8 | 7.4 | -- | -- |
| **Participation rate (%)** | 21.6 | 37.6 | 24.2 | 68.4 | 76 | 74.4 | 1.9 | 2.3 |

*Comparison of characteristics between complete respondents and adults in New York City based on the American Community Survey, 2020*

To assess whether there may have been bias due to different respondent characteristics, we compared the unweighted distributions of those completing the survey to that of adult NYC residents from the 2020 American Community Survey (ACS). We did not find large discrepancies between the survey sample and NYC population except that our survey respondents skewed slightly older, which is likely due to older adults being available to respond to the survey. While this suggests that the distribution of responders did not vastly differ from the distribution of the general NYC population, we cannot rule out if COVID-19 or vaccination status differed between survey responders and those who did not respond. Importantly, in our study, potential respondents were not informed in advance that the survey would be about COVID, but rather told that the survey was focused on public policy.

Supplementary Table 2

| ***Race/ethnicity*** | ***Survey Completers*** | ***ACS NYC 2020*** |
|---|---|---|
| *White NH* | *39.8* | *31.9* |
| *Black NH* | *20.7* | *21.4* |
| *Hispanic* | *28.5* | *28.9* |
| *AAPI* | *6.4* | *14.2* |
| *Other/more than one race* | *4.7* | *3.5* |

Supplementary Table 3

| ***Age*** | ***Survey Completers*** | ***ACS NYC 2020*** |
|---|---|---|
| *18-24* | *5.1* | *8.3* |
| *25-34* | *8.7* | *17.8* |
| *35-44* | *6.8* | *13.6* |

| | | |
|---|---|---|
| *45-54* | *8.8* | *12.5* |
| *55-64* | *17.0* | *12.1* |
| *65+* | *53.6* | *15.0* |

Supplementary Table 4

| ***Gender*** | ***Survey Completers*** | ***ACS NYC 2020*** |
|---|---|---|
| *Male* | *41.5* | *47.7* |
| *Female* | *55.1* | *52.3* |
| *Non-binary* | *3.5* | *--* |

Supplementary Table 5

| ***Education*** | ***Survey Completers*** | ***ACS NYC 2020*** |
|---|---|---|
| *Less than HS* | *12.2* | *16.7* |
| *HS graduate* | *21.4* | *23.7* |
| *Some college* | *25.2* | *22.4* |
| *College grad* | *41.2* | *37.1* |

## Supplementary Note 1

*Survey questionnaire*

***Survey on recent COVID exposure, COVID infection, and testing behaviors in New York City***

Hello, this is XYZ with a brief public policy survey. At no time will we try to sell you anything. We are just interested in your opinions, and you can drop out at any time.

To begin, what language would you like to take this survey in?
  a. English
  b. Español

**The following questions will ask about COVID exposure in the past 2 weeks**

1. In the past 2 weeks, have you experienced any COVID-like symptoms (e.g., 100 degrees fever or higher, chills, cough, sore throat, fatigue, headache, shortness of breath, congestion or runny nose, muscle aches, loss of smell or taste, nausea, or diarrhea)?
    a. Yes
    b. No
    c. Don’t know/not sure

2. In the past 2 weeks, has anyone in your household (not including yourself) experienced COVID-like symptoms or tested positive for COVID-19?
    a. Yes
    b. No
    c. Don’t know/not sure

3. In the past 2 weeks, were you exposed to any other person outside of your household who had COVID-like symptoms or tested positive for COVID-19?
    a. Yes
    b. No
    c. Don’t know/not sure

**The following questions will ask about COVID testing and treatment in the past 2 weeks**

4. In the past 2 weeks, have you taken an at-home rapid test for COVID-19? (a rapid at-home test allows you to collect your own sample and get results within minutes at home)
    a. Yes, Tested Positive
    b. Yes, Tested Negative
    c. No, I have not tested

5. In the past 2 weeks, have you taken a rapid antigen or PCR test for COVID-19 from a healthcare or testing provider?
    a. Yes, Tested Positive (go to 6)
    b. Yes, Tested Negative (go to 6)
    c. No, I have not tested (go to 9)

6. In the past 2 weeks, which of the following locations did you get tested for COVID-19?
   a. Hospital or physician’s office
   b. Urgent care clinic
   c. Pharmacy
   d. Mobile testing site
   e. Employer
   f. Other

7. In the past 2 weeks, how difficult was it for you to get yourself a viral COVID-19 test at a healthcare or testing provider (PCR or rapid) if you attempted to get one?
   a. Did not attempt to get a test
   b. Not Difficult
   c. Somewhat Difficult
   d. Very Difficult

8. In the past 2 weeks, did you try to get paxlovid, an antiviral medication for COVID-19?
   a. No, I don’t know about paxlovid
   b. No, I did not try to get a paxlovid prescription
   c. Yes, I received a paxlovid prescription
   d. Yes, I tried to get paxlovid, but was unable to get it

9. Have you ever self-administered an at-home rapid test for COVID-19 for yourself or for someone in your household?
   a. Yes
   b. No
   c. Don’t know/not sure

10. If you had easy access to free at-home rapid tests, would you prefer to test for COVID-19 at using at-home rapid tests or at a health care/test center?
    a. Take at-home rapid test
    b. Health Care/Test Center
    c. Don’t know/not sure

**Do you agree or disagree with the following statements about COVID-19 at-home testing?**

11. At-home rapid tests are easy to use:
    a. Strongly agree
    b. Agree
    c. Neither agree nor disagree
    d. Disagree
    e. Strongly disagree

12. I can easily get an at-home rapid test if I or someone in my household needs one:
    a. Strongly agree
    b. Agree
    c. Neither agree nor disagree
    d. Disagree
    e. Strongly disagree

13. Since March 2020, have you ever had COVID-19 infection and tested positive, either at home or with a provider?
    a. Yes, once
    b. Yes, more than once
    c. No, but I am pretty sure that I had COVID
    d. No, I never tested positive, and I don't think I have ever had COVID
    e. Don't know/not sure

14. Were you aware that New York City just moved from the low to the medium COVID-19 risk level, indicating higher levels of community transmission?
    a. Yes
    b. No
    c. Don't know/not sure

**Respondent Characteristics**

15. Do you currently have any kind of health care coverage, including health insurance, prepaid plans such as HMOs, or government plans such as Medicaid or Medicare, or Indian Health Service?
    a. Yes
    b. No
    c. Don't know/not sure

16. Do you have any of the following conditions that could increase the severity of COVID-19: cancer, diabetes, obesity, COPD or lung disease, liver disease, heart disease, high blood pressure, a recent organ transplant, or an immunodeficiency)?
    a. Yes
    b. No
    c. Don't know/not sure

17. 17. Have you been fully vaccinated against COVID-19? [Either 2 doses of mRNA vaccine series (Moderna or Pfizer) or a single dose of Johnson and Johnson COVID-19 vaccine]
    a. Yes (go to 18)
    b. No (go to 19)
    c. Don't know/not sure (go to 19)

18. If you have been fully vaccinated, have you also received a coronavirus booster?
    a. Yes, more than 5 months ago
    b. Yes, within the past 5 months
    c. No

19. If not fully vaccinated OR not boosted: Do you plan to get a vaccine dose or booster in the next two weeks?
    a. Yes
    b. No
    c. Don't know/not sure

20. Which zip code do you reside in?

21. What is your age?

a. 18-24
b. 25-34
c. 35-44
d. 45-54
e. 55-64
f. 65-74
g. 75 +

22. How do you currently identify your gender? Do you identify as …
    a. Male
    b. Female
    c. Gender non-binary

23. Are you Latino/a, or of Hispanic or Spanish origin?
    a. Yes
    b. No

24. Which one of the following would you use to describe yourself ?
    a. White
    b. Black or Black American
    c. Asian, Native Hawaiian or Other Pacific Islander
    d. More than one race

25. Were you born in the USA [Puerto Rico and other territories are considered outside the USA]?
    a. Yes
    b. No
    c. Don't know

26. What is the highest grade or year of school you completed?
    a. Less than high school
    b. Grade 12 or GED (High school graduate)
    c. College 1 year to 3 years (Some college or technical school, associate degree)
    d. College 4 years or more (College graduate)

27. How many members of your household, including yourself, are 18 years of age or older?
    a. 1
    b. 2
    c. 3
    d. 4
    e. 5
    f. 6
    g. 7
    h. 8
    i. 9 Or more

28. How many children 17 years old or younger usually live or stay with you?
    a. 0
    b. 1

c. 2
d. 3
e. 4
f. 5
g. 6
h. 7
i. 8
j. 9 Or more

29. Are you currently employed for wages or salary?
   a. Yes
   b. No
   c. Don’t know/not sure

30. What is your household’s annual income?
   a. $25,000 or less
   b. Between $25,001 - $65,000
   c. Between $65,000 - $150,000
   d. Above 150,000
   e. Refuse
